# Molecular characterization of LASP-1 expression reveals vimentin as its new partner in human hepatocellular carcinoma cells

**DOI:** 10.3892/ijo.2015.2923

**Published:** 2015-03-09

**Authors:** ALESSANDRO SALVI, ITALIA BONGARZONE, LIA FERRARI, EDOARDO ABENI, BRUNA ARICI, MAIDA DE BORTOLI, SABRINA SCURI, DANIELA BONINI, ILARIA GROSSI, ANNA BENETTI, GIANLUCA BAIOCCHI, NAZARIO PORTOLANI, GIUSEPPINA DE PETRO

**Affiliations:** 1 Department of Molecular and Translational Medicine, Division of Biology and Genetics, University of Brescia, Brescia, Italy; 2 Department of Experimental Oncology and Molecular Medicine, Proteomics Laboratory, Fondazione IRCCS Istituto Nazionale dei Tumori, Milan, Italy; 3 Department of Clinical and Experimental Sciences, Surgical Clinic, University of Brescia, Brescia, Italy; 4 Department of Clinical and Experimental Sciences, Division of Morbid Anatomy, University of Brescia, Brescia, Italy

**Keywords:** hepatocellular carcinoma, LASP-1, vimentin, mass spectrometry, qPCR

## Abstract

Hepatocellular carcinoma (HCC) is the third most common cause of cancer-related mortality worldwide. We have previously reported that LASP-1 is a downstream protein of the urokinase type plasminogen activator (uPA). Here we investigated the role of LASP-1 in HCC by a molecular and biological characterization of LASP-1 expression in human HCC specimens and in cultured HCC cells. We determined the LASP-1 mRNA expression levels in 55 HCC cases with different hepatic background disease. We identified 3 groups of patients with high, equal or low LASP-1 mRNA levels in HCC tissues compared to the peritumoral (PT) tissues. In particular we found that i) the HCCs displayed a higher LASP-1 mRNA level in HCC compared to PT tissues; ii) the expression levels of LASP-1 mRNA in female HCCs were significantly higher compared to male HCCs; iii) the cirrhotic HCCs displayed a higher LASP-1 mRNA. Further, the biological characterization of the ectopic LASP-1 overexpression in HCC cells, using MALDI-TOF mass spectrometer on the LASP-1 co-immunoprecipitated fractions, displayed vimentin as a novel putative partner of LASP-1. Our results suggest that LASP-1 mRNA overexpression may be mainly implicated in female HCCs and cirrhotic HCCs; and that LASP1 may play its role with vimentin in HCC cells.

## Introduction

Hepatocellular carcinoma (HCC) ranks as the fifth most common malignant disorder and the third leading cause of cancer-related deaths worldwide (1). The most important causes leading to HCC are the HBV and HCV infections, heavy alcohol consumption, aflatoxin B1, gender (males are more susceptible than females), obesity associated with non-alcoholic fatty liver disease, and α1-antitrypsin deficiency (2). Surgical resection, liver transplantation and ablative procedures are considered the curative procedures especially for early-stage HCC but also for these patients, bearing the most favorable conditions, the final prognosis is not completely satisfactory (3). The increasing understanding of the tumor biology of HCC could be helpful for the development of future targeted HCC therapeutics. The basic research community has to pursue this objective to include into the clinical practice some new molecular markers reflecting the biological aggressiveness of HCC.

LIM and SH3 protein 1 (LASP-1) was initially identified from a cDNA library of metastatic axillary lymph nodes of breast cancer patients. The human gene is located on 17q21 chromosome, it encodes a protein of 261 amino acids containing an N-terminal LIM domain followed by two actin binding domains in the core of the LASP-1 protein mediating an interaction between LASP-1 and the actin cytoskeleton (4,5). The SH3 domain at the C-terminus is involved in protein-protein interactions specifically with zyxin, pallidin, lipoma preferred partner (LPP) and vasodilator-stimulated phosphoprotein (VASP). The exact functions of LASP-1 are still not completely elucidated. LASP-1 is localised in dynamic actin assembly such as focal contacts, focal adhesions, lamellipodia membrane ruffles and pseudopodia where it interacts with motility-associated proteins and functions as structural scaffold (6–8).

LASP-1 is overexpressed in several human malignancies included human metastatic breast cancer, ovarian cancer, colorectal cancer, malignant childhood medulloblastoma, hepatocellular carcinoma, bladder and oral cancer, and prostate carcinoma (9–15). *In vitro* studies showed that LASP-1 plays an important role in tumor development and metastases. The knock-down of LASP-1 by RNA interference resulted in a strong inhibition of the proliferation and migration of various cancer cells, such as breast, ovarian, colorectal and prostate cancer cell lines (9,15,16). In certain types of malignant cells the nuclear localization of the protein was observed. LASP-1 expression and nuclear localization correlated significantly with tumor size, nodal positivity and a poor long-term survival of the patients affected by breast cancer (17–19). In a previous study, we found that LASP-1 is a downstream protein of the urokinase type plasminogen activator (uPA) and its mediator in HCC cell migration likely taking part in the cytoskeleton changes that occur during this process (20). Ectopic uPA overexpression induced LASP-1 upregulation and cell motility in HCC cells. However, ectopic LASP-1 overexpression did not upregulate uPA expression. In the present study, we investigated the biological role of LASP-1 in HCC by a molecular and biological characterization of LASP-1 expression in human HCC specimens and in cultured HCC cells. We ascertained the heterogenous expression level of LASP-1 mRNA in HCC with different hepatic background disease and we have biologically characterized the ectopic LASP-1 overexpression in HCC cells.

## Materials and methods

### Cell cultures

SKHep1Clone3 (SKHep1C3), SKHep1C3 nod.69.2, selected from human HCC-derived cells (SKHep1: ATCC HTB-52), AB15 and AB19 human dermal fibroblasts were maintained in Earle’s MEM (Life Technologies, Carlsbad, CA, USA) supplemented with 10% foetal bovine serum (Life Technologies) at 37°C in a 5% CO_2_ incubator. Differentiated human HCC-derived cells (HepG2, ATTC HB-8065; HuH-6; HuH7) and HA22T/VGH undifferentiated HCC-derived cells were maintained in RPMI-1640 (Life Technologies) supplemented with 10% foetal bovine serum and 1 mM sodium pyruvate at 37°C in a 5% CO_2_ incubator. The HuH-6 and HA22T/VGH cells were kindly provided by N. D’Alessandro (University of Palermo, Italy).

### Tissues and clinicopathological features of HCC and evaluation of LASP-1 expression in tumoral and peri-tumoral (PT) human tissues by qPCR

All human HCC samples (n=55) as well as the corresponding PT non-tumor samples (resected 1–2 cm from the malignant tumor) were obtained from HCC patients for pathological examination. Each biopsy specimen was obtained with the patient’s informed consent under standard conditions of sampling and processing (40). Each specimen was determined to be HCC or PT by pathological examination. In this study, 55 HCC subjects underwent surgical resection. The subjects consisted of 35 men and 20 women (54 Italian and 1 Chinese) ranging from 38 to 82 years of age (mean age: 68.7±8.4 years). The subjects did not have any apparent distant metastases, and none had been previously treated for HCC. We subdivided the cases on the basis of presence or absence of liver cirrhosis (31 HCC with cirrhosis, 24 HCC without cirrhosis). Twenty-three patients were HCV positive, 12 were HBV positive, 3 were both HBV and HCV positive, and 16 were both HBV and HCV negative, for 1 patient no information was available (Table I). The total RNA from tissue samples was isolated using TRIzol reagent (Invitrogen), according to the manufacturer’s instructions. The expression of LASP-1 mRNAs in the tissues was evaluated using TaqMan Gene Expression Assay (Applied Biosystem). GAPDH was used as an internal standard.

The PCR mixture (25 μl) containing 1 μl of the specific probe, 11.25 μl of cDNA and 13.75 μl of Taq-Man 2X Universal PCR Master Mix were incubated in a 7500 Applied Biosystems instrument initially at 55°C for 2 min, then at 95°C for 10 min, followed by 40 cycles of 95°C for 15 sec and 60°C for 60 sec. The expression of LASP-1 mRNAs (RQ) was based on the ΔΔC_T_ method.

For each case the ratio (R) between the relative levels in HCC (RQ_HCC_) and those in PT (RQ_PT_) was calculated. The mRNA expression level was considered to be decreased for a R-value ≤0.8 and increased for a R-value ≥1.2. A value between 0.8 and 1.2 was defined as having no change in expression level.

### Immunohistochemical analysis

Tissue sections (5 μm) were de-paraffinized in xylene, rehydrated in ethanol, incubated in 0.3% H_2_O_2_ in methanol for 20 min to block endogenous peroxidase activity; 3% BSA was used to block non-specific staining. The sections were washed with 1X PBS and incubated with rabbit anti-human LASP-1 (1:50 v/v) overnight (Santa Cruz Biotechnology, Biosource, CA, USA). The biotinylated secondary antibodies were added for 15 min (Super Sensitive IHC Detection Systems, BioGenex, San Ramon, CA, USA). After extensive washing, the sections were incubated with horseradish peroxidase complex (ABC complex) for 15 min. The chromogen DAB was used to localize the peroxidase in tissues. The slides were counterstained with H&E and analyzed with an optical microscope at ×10, ×40 and ×60 magnification.

### Lasp-1 cloning and transient transfection of HA22T/VGH with pGPF-LASP1

The full lenght coding region (CDS) of LASP-1 was amplified from SKHep1C3 cDNA using the LASP-1 cl Forward: 5′-ggaaccatgaaccccaac-3′ and SH3 Reverse: 5′-cctccacgtagttggccg-3′ primers and then directly cloned in the pcDNA3.1-CT-GFP-TOPO vector (Life Technologies) upstream the GFP gene following manufacturer’s instructions. The correct sequence and the orientation of the insert were ascertained by direct automatic sequencing of the plasmid. The vector, named pGFP-LASP1, was transiently transfected in HA22T/VGH cells. Briefly, 2,900,000 HA22T/VGH cells were seeded in 10-cm diameter Petri dishes and 24 h later, when the cells reached 80% confluency, they were transfected with 24 μg/dish of pGFP-LASP1 plasmid using 60 μl/dish of Lipofectamine (Life Technologies) following the manufacturer’s instructions. After 72 h of transfection, cell lysates were collected for immunoprecipitation analysis in NP-40 buffer (Life Technologies) containing phosphatase (Sigma) and protease (Roche) inhibitor cocktails or in 0.05% SDS for routine analysis by western blotting (WB).

### LASP-1-GFP immunoprecipitation

Protein G Dynabeads^®^ (50 μl) (Life Technologies) per sample were incubated overnight at 4°C with 10 μg of mouse monoclonal anti-human LASP-1 (Chemicon International) or mouse monoclonal anti-human vimentin (Santa Cruz Biotechnology) or mouse IgG1 (negative control). The day after the Ab-Dynabead complexes were incubated overnight with cell extracts from pGFP-LASP1 transfected HA22T/VGH or HA22T/VGH control cell extracts in NP-40 buffer (1 ml, containing ~1 mg of total proteins). The day after, the Ag-Ab-Dynabead complexes were placed on the magnet and the supernatants were kept for further analysis (immunodepleted = ID). After 3 washes of the Ag-Ab-Dynabeads with washing buffer, 30 μl of elution buffer per sample were added and the complex was incubated at room temperature for 2 min. The Dynabeads were separated on the magnet and the supernatants containing the Ab-Ag complex (IP CTRL, IP α-LASP-1) were placed in a clean tube for proteomic analysis.

### Proteomic identification of LASP-1 partner proteins

Prior to the mass spectrometry (MS) analysis and in order to confirm that we had obtained precipitated proteins, we analyzed aliquots of our samples on 4–12% gels by sodium dodecyl sulfate-polyacrylamide gel electrophoresis (SDS-PAGE) and Western blotting according to the manufacturer’s instructions.

The IP CTRL and IP α-LASP-1 and the corresponding antibodies were loaded on one-dimensional 4–12% NuPAGE^®^ precast gels (Life Technologies). Proteins were visualized with G250 Coomassie Blue (Bio-Rad) or with silver staining by standard procedures. For protein profiling, protein bands were excised from Coomassie-stained preparative gels and processed as previously described (42). MALDI-TOF-MS was carried out using a Voyager-DE STR (Applied Biosystems), equipped with a nitrogen laser (337 nm). Monoisotopic peptide masses were analyzed using the Aldente software http://www.expasy.org/tools/webcite. The input was searched according to: Aldente, UniProtKB/SwissProt; predefined taxon, Mammalia; Spectrometer internal error max, 25. Only proteins identified in two separate experiments were considered.

### Western blot analysis

The samples were electrophoresed in 4–12% Bis-Tris gels at 100 V using MES running buffer. The proteins were electrophoretically transferred to a nitrocellulose membrane at 100 V for 1.5 h. The membrane was blocked with 3% milk in PBS 1X at 37°C for 2 h. The blots were incubated with primary antibodies: rabbit anti human LASP-1 polyclonal antibody, 1:500 in 0.3% BSA-PBS (Millipore); rabbit anti-green fluorescent protein polyclonal antibody, 1:100 in 0.3% BSA-PBS (Santa Cruz Biotechnology); mouse anti-vimentin monoclonal antibody, 1:1000 in 0.3% BSA-PBS (Santa Cruz Biotechnology) at room temperature overnight, washed three times with PBS, then incubated with an alkaline-phosphatase-conjugated secondary antibody (1:7500 in 0.3% BSA-PBS) for 4 h at 37°C. The results of the immunoreaction were detected with Nitroblue tetrazolium and bromochloroindolyl phosphate (Promega).

### Immunofluorescence and confocal immunofluorescence analysis

For the immunofluorescence detection of GFP, HA22T/VGH were seeded and cultured (75,000 cells/22×22 mm glass coverslips in 3-cm diameter Petri dishes) in growth medium. After 24h the cells were transfected with pGFP-LASP1 as described above and after 48h the cultures were fixed in cold methanol × 20 min at 4°C. The coverslips were directly mounted on glass slides in mounting medium and photographed with a Leitz Fluorescence microscope (magnification, ×63).

For the immunofluorescence detection of vimentin, HA22T/VGH were seeded and cultured (75,000 cells/22×22 mm glass coverslips in 3-cm diameter Petri dishes) in growth medium. After 24 h the cells were fixed in cold methanol ×20 min at 4°C, after one washing in PBS (1×3 min) and one treatment with 0.3% BSA in PBS (1×3 min) the cells were immunoreacted with the first antibody, mouse monoclonal anti-vimentin (1:50 in 0.3% BSA) for 1 h at room temperature and washed 3×5 min in PBS. The cells were then immunoreacted with the secondary rhodamine-conjugated anti-mouse IgG (1:100 in 0.3% BSA) (Calbiochem, San Diego, CA, USA) for 30 min at room temperature. The coverslips were mounted on glass slides in mounting medium and photographed with a Leitz fluorescence microscope (magnification, ×63).

For the confocal microscopy, AB15 and HA22T/VGH cells were grown, fixed and permeabilized as described for immunofluorescence microscopy. The samples were incubated with primary polyclonal antibody against LASP-1 and with primary monoclonal antibody against VIM (1:100 in 0.3% BSA) for 30 min at room temperature. After the last washing step, the unconjugated primary antibodies were recognized with secondary antibodies (1:400 in 0.3% BSA) conjugated with Alexa-Fluor 555 and Alexa-Fluor 488 dyes (Life Technologies, Invitrogen) for 30 min at RT. DNA was counterstained with DAPI (1:3000) (Calbiochem) for 10 min. The coverslips were mounted on glass slides in SlowFade Gold antifade reagent (Life Technologies, Invitrogen). The images were acquired on Zeiss LSM 510 Meta confocal microscope (Carl Zeiss, Milan, Italy).

### Statistical analysis

**Figure 1:**
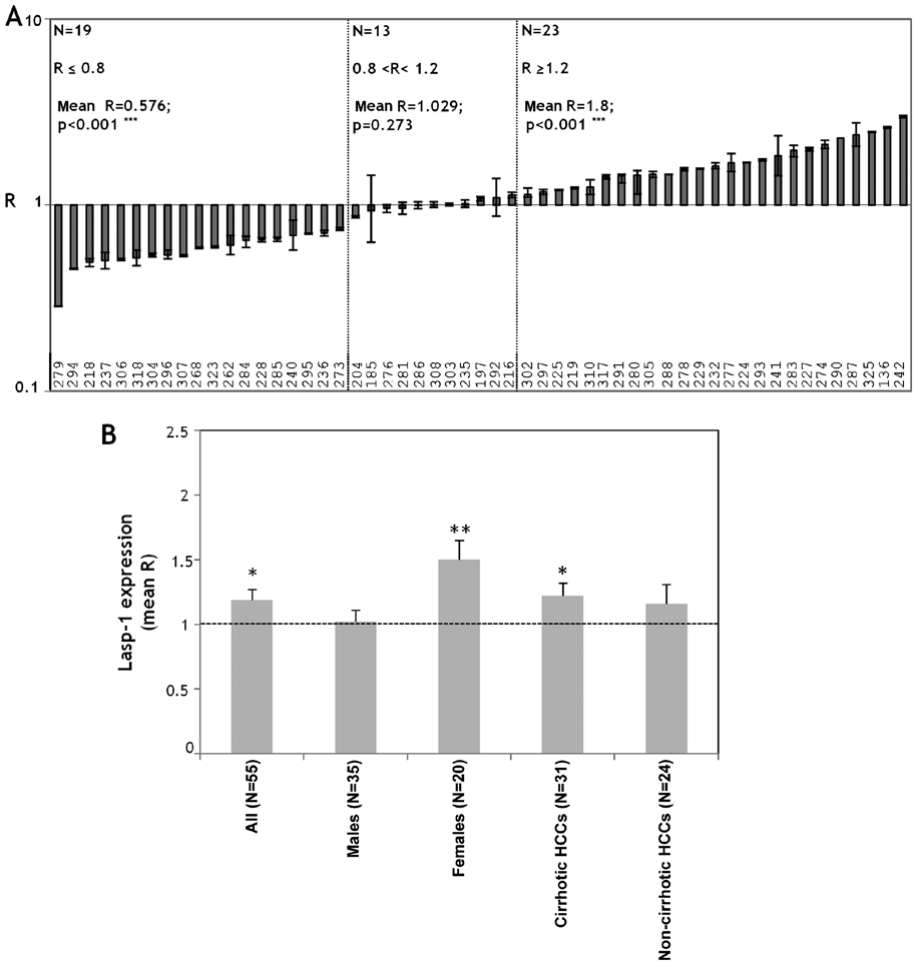
The expression of LASP-1 mRNA by qPCR in HCC and PT tissues from the human biopsies of patients affected by HCC. (A) The R-values of the 55 cases examined are reported. The R-values are the ratio between the RQ (relative quantification) of LASP-1 mRNA expression in HCC vs. PT of each case. We assumed LASP-1 upregulation when R≥1.2, LASP-1 downregulation when R≤0.8 and no variation when 0.8<R<1.2 and consequently the cases can be subdivided into 3 groups: R≤0.8 (n=19); 0.8<R<1.2 (n=13); R≥1.2 (n=23). Histograms represent R, bars are RQmin_(HCC/PT)_; RQmax_(HCC/PT)_. (B) Mean R of LASP-1 mRNA in HCCs and the PTs tissues of all cases considered, male and female HCC patients and cirrhotic and non-cirrhotic HCCs. Histograms represent mean R, bars are SEM. ^*^p<0.05; ^**^p<0.01; ^***^p<0.001.

Each experiment was carried out at least twice. The histograms represent the mean values, and bars indicate standard errors (SE) of the mean. For the data shown in Fig. 1 statistical significance of the results was determined using Student’s t-test for single group mean (expected value=1) and data were considered significant when P≤0.05. Statistical analysis was performed with kyplot, version 2.0 beta 13 (http://www.woundedmoon.org/win32/kyplot.html).

## Results

### LASP-1 mRNA is significantly overexpressed in HCC, particularly in female HCC patients and in cirrhotic HCCs

The expression levels of LASP-1 mRNA were evaluated by qPCR in 55 pairs of HCC and PT tissues. The R-value for each sample was the ratio between the LASP-1 expression level in the HCC tissue (RQ_HCC_) and the expression level detected in the relative non-tumor counterpart PT (RQ_PT_). We arbitrary assumed LASP-1 upregulation when R≥1.2, LASP-1 downregulation when R≤0.8 and no variation when 0.8<R<1.2. So doing the cases were subdivided into 3 groups: R≥1.2 (n=23); R≤0.8 (n=19); 0.8<R<1.2 (n=13) (Fig. 1A). Thus, the first group was defined by higher LASP-1 mRNA levels in HCC than PT (mean R=1.8; p<0.001); the second group was characterized by lower LASP-1 mRNA levels in HCC than PT (mean R=0.576, p<0.001); the third group was defined by equal levels of LASP-1 mRNA in HCC and PT tissues (mean R=1.029). No clinic parameter correlated with the LASP-1 mRNA expression (gender, age, tumor grading, background hepatic disease, viral hepatitis infection) in the 3 groups. Considering the mean LASP-1 expression in all the HCCs and PTs (N=55) we evidenced increased levels in HCC tissues compared to PTs (mean R=1.20, p<0.05) (Fig. 1B). The female HCC patients (N=20) overexpressed LASP-1 mRNA in HCC tissues (mean R=1.5, p<0.01) (Fig. 1B); the males (N=35) did not show the disregulation of LASP-1 mRNA between HCC and PT tissues (mean R=1.02) (Fig. 1B). The statistical analysis performed by using the Mann-Whitney test for unpaired data between mean R-values of males and females showed a significant differential expression of LASP-1 mRNA in male and female HCC patients (p=0.0147).

We further stratified the cases on the basis of the presence (31/55) or absence (24/55) of cirrhosis as a background liver disease. LASP-1 expression levels increased in HCCs compared to PTs in the cirrhotic cases (mean R=1.22, p<0.05) (Fig. 1B). No change in LASP-1 expression levels was observed in non-cirrhotic cases (mean R=1.15) (Fig. 1B).

### Levels of LASP-1 protein and mRNA expression are comparable and they increase in recurrent HCC

To compare LASP-1 mRNA and protein expression levels and to ascertain its cellular localization in some samples, immunohistochemistry (IHC) was performed in paired HCC tumors and their matched adjacent non-tumor tissues. We did not include all the samples because, during our work, IHC evaluation of LASP-1 protein expression was assessed by other authors (12). The tissues analyzed here were obtained from the biopsy specimens of 4 different HCC patients. Three cases (LV 227, LV 228, LV 229) were selected among the 55 cases analyzed for LASP-1 mRNA expression, 1 case (LV 144) was selected since the primary tumor and the intra-hepatic tumor recurrence tissues were available (the intrahepatic recurrent HCC was developed 28 months after the primary tumor resection). The LASP-1 protein expression level reflected the LASP-1 mRNA levels in the cases examined.

**Figure 2:**
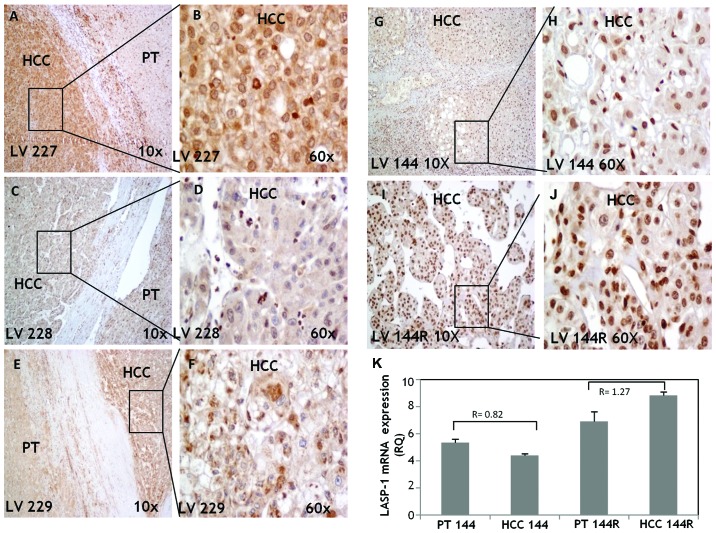
The evaluation of LASP-1 protein expression in HCC and PT tissues by IHC. LV 227. Strong LASP-1 expression in HCC, weak expression of LASP-1 in adjacent non-tumors tissue (A). Higher magnification shows positive cytosolic and weak nuclear expression of LASP-1 in HCC (B). LV 228. In PT tissue LASP-1 expression was detectable only in the inflammatory cells (C); weak cytoplasmic and nuclear expression of LASP-1 in HCC (D). LV 229. Weak cytoplasmic positivity of LASP-1 in PT, moderate expression in HCC (E); strong cytoplasmic and weak nuclear staining in HCC tissue (F). LV 144. Moderate nuclear staining particularly in differentiated area of HCC tissue (G and H). LV 144 recurrence. Strong nuclear positivity in this moderate differentiated HCC, negative or very weak cytoplasmic positivity (I and J). LASP-1 mRNA expression levels evaluated by qPCR in LV144 and LV144R (K). Histograms represent RQ (relative quantification) values, bars are ± RQ_max_, RQ_min_.

In LV 227 and LV 229 (Fig. 2A and B, E and F, respectively) LASP-1 protein was greatly upregulated in HCC compared to PT with a cytoplasmic staining and weak/moderate nuclear positivity. In LV 228 (Fig. 2C and D) LASP-1 was essentially detectable in the inflammatory cells of the PT tissues while in HCC LASP-1 was weakly expressed in the cytoplasm and nuclei. LV 144 displayed moderate nuclear staining, particularly in differentiated areas (Fig. 2G and H); its intra-hepatic metastasis LV 144R (Fig. 2I and J) showed LASP-1 strong nuclear staining and very weak cytoplasmic positivity. LASP-1 mRNA levels were higher in PT 144R and HCC 144R than PT 144 and HCC 144 and the LASP-1 level in HCC 144R increased 2 fold compared to HCC 144 (RQ_HCC144R_=8.821; RQ_HCC144_=4.402) (Fig. 2K).

### Immunoprecipitation of LASP-1-GFP fused protein and MALDI-TOF-MS identification of its molecular interactors in HA22T/VGH cells

**Figure 3:**
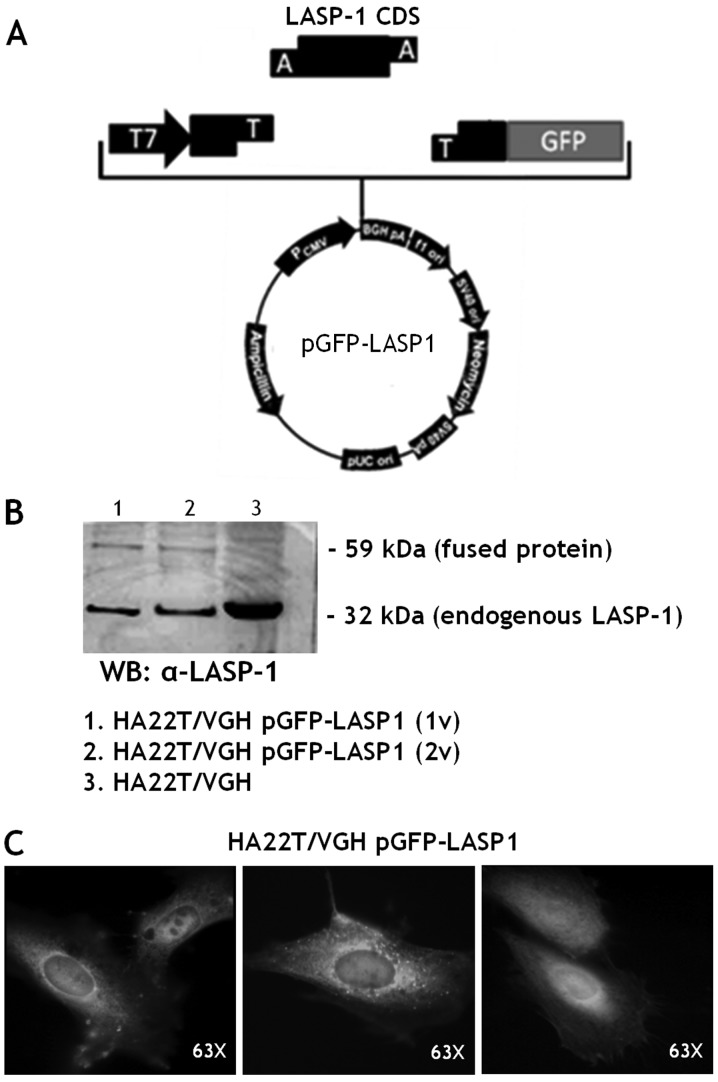
(A) Cloning and expression of the fused protein LASP-1-GFP. The LASP-1 CDS was cloned upstream the GFP gene in a TA-plasmid with the strong promoter CMV and containing the ampicillin and neomycin selectable marker genes. (B) The HA22T/VGH cells were transiently transfected with the plasmid pGFP-LASP1 and the cell extracts were evaluated for the endogenous LASP-1 expression of (32 kDa) and for the fused protein (59 kDa) by western blotting (WB) using anti-LASP-1 (1v=1 volume; 2v=2 volumes). (C) Expression and localization of the LASP-1-GFP fused protein in HA22T/VGH transfected cells; magnification, ×63.

LASP-1 CDS was cloned upstream the GFP coding gene in the pCDNA3.1 expression vector (Fig. 3A). The plasmid, named pGFP-LASP1, was sequenced and then transiently transfected in the HA22T/VGH cell line (HA22T/VGH pGFP-LASP1).

The fused protein (59 kDa) was detected in the transfected cells while the endogenous LASP-1 (32 kDa) was detected in transfected and untransfected HA22T/VGH cells (Fig. 3B) as expected. The fluorescent fused protein LASP-1-GFP was directly observed under a fluorescence microscope. It was mainly localized in the cytoplasm and in the peri-nuclear area of the HA22T/VGH cells (Fig. 3C).

**Figure 4:**
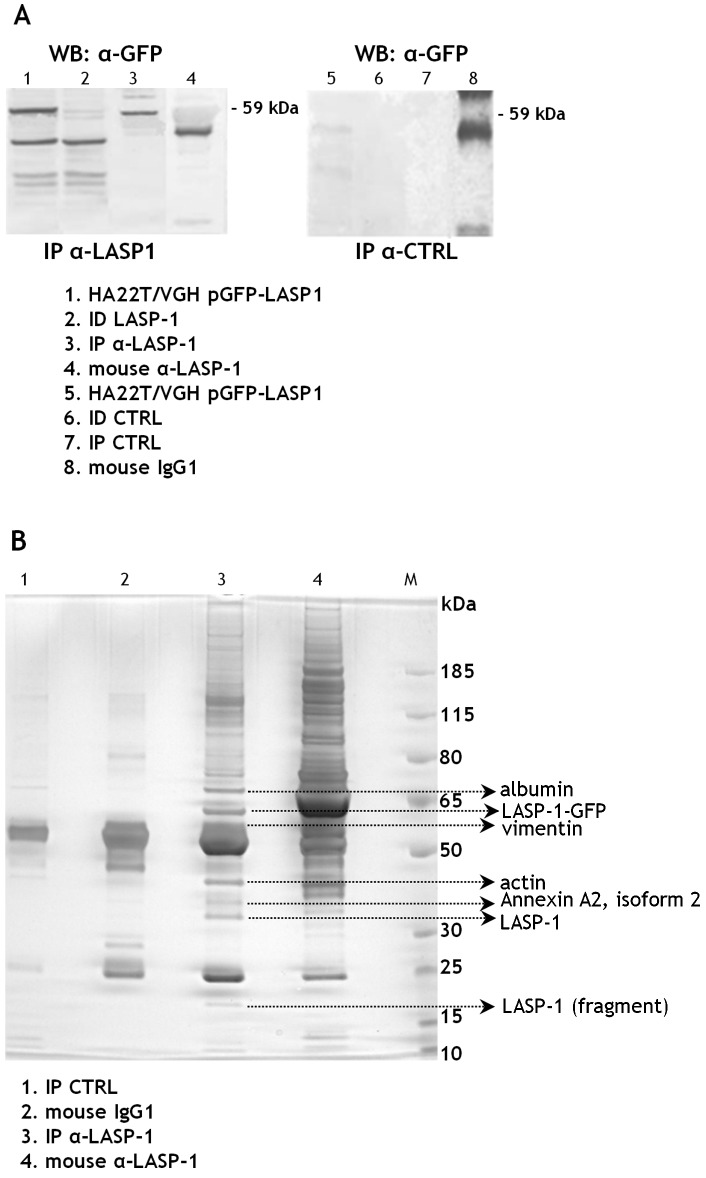
The identification of LASP-1 binding partners. (A) The fused protein LASP-1-GFP was immunoprecipitated in HA22T/VGH cells using mouse monoclonal anti-LASP-1 antibodies. The fused protein (59 kDa) was detected in transfected cells (lane 1) and in IP α-LASP-1 (lane 3) by western blotting (WB) using anti-GFP antibodies. The mouse immunoglobulins (IgG1) were used to obtain a negative IP control (IP CTRL, lane 7). The antibodies used for immunoprecipitation were also loaded (lanes 4 and 8); (IP=immunoprecipitated; ID=immunodepleted). (B) Silver staining of LASP-1-GFP associated proteins in HA22T/VGH cell lysate immunoprecipitated with anti-LASP1 (IP α-LASP-1: lane 3) and with control mouse immunoglobulins (IP CTRL: lane 1) and separated by SDS-PAGE. The antibodies used for immunoprecipitation were also loaded (lanes 2 and 4). Arrows mark the protein bands cut off and identified by mass spectrometry analysis in both the experiments performed.

To identify new molecular partners of LASP-1 in HCC cells we first immunoprecipitated the proteins from the cell extracts of the transfected HA22T/VGH cells using the mouse monoclonal anti-LASP-1 antibodies immobilized on magnetic beads. The presence of the fused 59 kDa protein in the LASP-1 immunoprecipitated fraction (IP α-LASP1) was tested by WB with anti-GFP antibodies (Fig. 4A). No bands were detected in the control cell extracts immunoprecipitated with the mouse IgG1 (IP CTRL) and this demonstrated the absence of proteins aspecifically bound to the mouse antibody used or to the magnetic beads.

The LASP-1 (IP α-LASP1), the control (IP CTRL) immunoprecipitated fractions and the antibodies employed to immunoprecipitate were separated on a bidimensional polyacrylamide gel. The differential protein bands present in the IP α-LASP1 fraction were excised and analyzed with MALDI-TOF-MS. The experiment was performed twice and vimentin (VIM) was detected in IP α-LASP1 both times with statistically significant scores. The same for actin, albumin and annexin (Fig. 4B).

**Figure 5:**
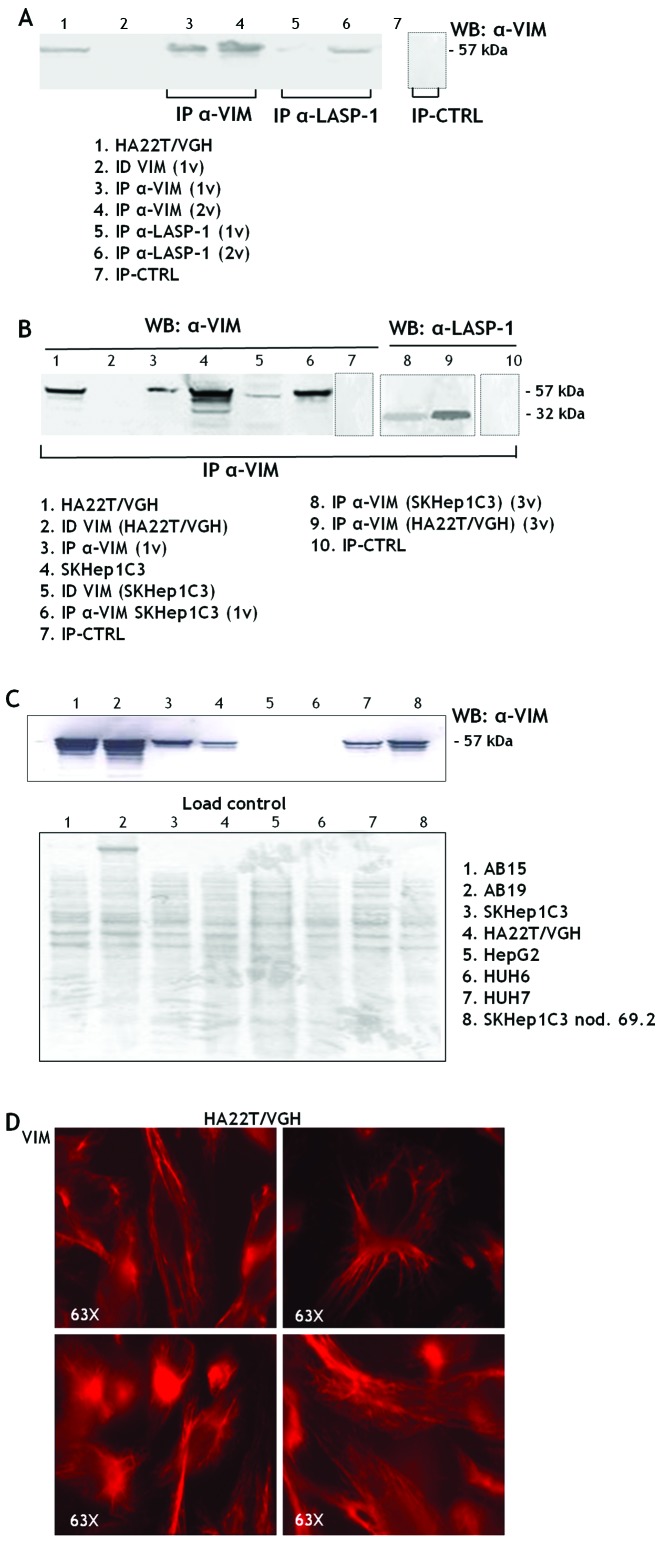
The validation of VIM as molecular partner of LASP-1. (A) The proteins from cell extracts of HA22T/VGH cells were immunoprecipitated with anti-LASP-1 and anti-VIM (IP α-VIM). VIM (57 kDa) was detected by WB in the IP α-VIM (lanes 3 and 4) and in IP α-LASP-1 (lanes 5 and 6) (1v=1 volume, 2v=2 volumes), it was not detected in the negative control (IP-CTRL). The boundaries of individual panels are evidenced with dashed line. (B) VIM was detected in IP α-VIM of the HA22T/VGH cells (lane 3) and SKHep1C3 cells (lane 6). LASP-1 was detected in IP α-VIM of the HA22T/VGH and SKHep1C3 cells (lanes 8 and 9) (3v=3 volumes). No bands were detected in the negative controls (IP-CTRL). The boundaries of individual panels are evidenced with dashed line. (C) Expression of VIM evaluated by WB in cells, extracts of human fibroblasts AB15 and AB19 (lanes 1 and 2) and in HCC cells (lanes 3–8) and loading control. (D) Evaluation of VIM expression in HA22T/VGH cells by immunofluorescence analysis.

To validate vimentin as a new molecular partner of LASP-1 we immunoprecipitated the proteins from HA22T/VGH cell extracts with anti-VIM and anti-LASP1 antibodies. The VIM protein (57 kDa) was detected in the IP α-VIM and in the IP α-LASP1 of the HA22T/VGH cells (Fig. 5A); the LASP-1 protein was detected in the IP α-VIM of both HA22T/VGH and SKHep1C3 cells (Fig. 5B, lanes 8 and 9); this demonstrates that VIM could be a molecular partner of LASP-1 in both HCC cell lines. VIM, a member of the intermediate filaments, is variably expressed in human HCC cells, in particular at high levels in the undifferentiated HCC cells (HA22T/VGH, SKHep1C3, SKHep1C3 nod. 69.2) and in fibroblasts (AB15 and AB19) (Fig. 5C). Like other cells of connective tissue, fibroblasts are derived from the primitive mesenchyme. Thus, they express the intermediate filament protein VIM. In more differentiated HCC cells VIM is not expressed (HepG2, HuH6) or it is expressed at lower levels (HuH7). The immunofluorescence analysis performed in HA22T/VGH cells revealed a cytoskeletal localization of VIM (Fig. 5D).

**Figure 6:**
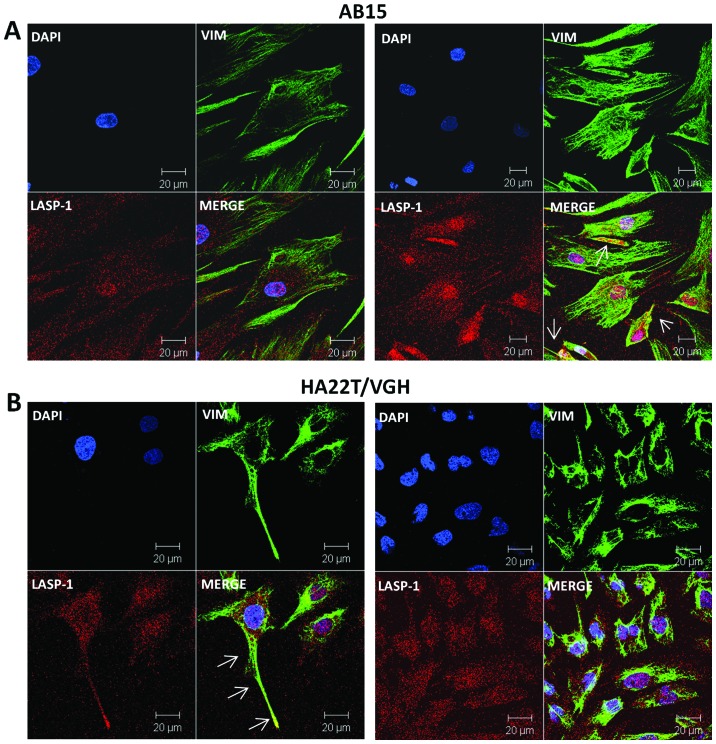
The confocal immunofluorescence analysis of LASP-1 (red) and VIM (green) co-localization in human cells. LASP-1 is localized in the nucleus (blue) and in membrane of the fibroblasts AB15 as evidenced by analysis of a vertical z-stack of 2 fields (A). VIM and LASP-1 co-localized in the cytoplasm as highlighted by white arrows. (B) VIM and LASP-1 co-localized in HA22T/VGH cells in particular at the filopodia and in the cytoplasm. Scale bar, 10–20 μm.

We further analyzed the eventual co-localization of VIM and LASP-1 in human cell lines, dermal fibroblasts (AB15) and HCC derived cells (HA22T/VGH) by confocal immunofluorescence. The Z-stack observation evidenced that LASP-1 in AB15 cells was localized both in the membrane and in the nuclei (Fig. 6A) and that VIM is mainly localized in the cytoplasm probably in the cytoskeleton structure. VIM and LASP-1 co-localized in AB15 cells as shown by white arrows (Fig. 6A, right). In HA22T/VGH cells LASP-1 is localized into the nucleus and in the cytoplasm and VIM in the cytoplasm (Fig. 6B). VIM and LASP-1 are co-localized in several portions of the cytoplasm and this supported the results obtained by co-immunoprecipitation experiments. VIM and LASP-1 also co-localized in the cellular extensions of HA22T/VGH (white arrows).

## Discussion

LASP-1 is a key protein that explicates its functions in the lamellipodia, filipodia, pseudopodia and focal adhesions during cell movement and it contributes in maintaining the cytoskeleton architecture by binding actin filaments (21). In various cancers, LASP-1 is overexpressed, it influences the aggressive behavior of the cells promoting proliferation, migration, invasion and metastasis (9–13). Concerning LASP-1 and HCC, it is known from global transcriptional profiles of matched pairs of HBV associated HCC tumor and non-tumor liver tissue specimens that LASP-1 is a gene significantly upregulated in this subgroup of HCC (22,23). The same authors reported qPCR validation data showing LASP-1 upregulation in 8/8 HBV associated HCC cases and further demonstrated that LASP-1 is transcriptionally repressed by p53 (23).

During the accomplishment of our study Wang *et al* (12) demonstrated the overexpression of LASP-1 protein in HCC; they showed no correlation between LASP-1 protein expression and liver cirrhosis of HCC patients while they found correlation between cytosolic and nuclear LASP-1 protein expression with hepatitis B surface antigen (HBsAg) of HCC patients. In this regard it is known that HBx could upregulate LASP-1 through PI3-K (24). In recent years, using a proteomics analysis, we found that LASP-1 is a mediator of uPA during migration of HCC cells (20) and that LASP-1 expression is coordinated with the overexpression of uPA, a negative prognostic marker for this type of cancer (25). In the present study, we wanted to investigate further the role of LASP-1 expression in HCC by determining for the first time the expression levels of LASP-1 mRNA in HCC tumors with different hepatic background disease and by characterizing the ectopic LASP-1 overexpression in HCC cells using MALDI-TOF mass spectrometry.

From the determinations of LASP-1 expression levels in all cases of HCC tested, LASP-1 mRNA levels were generally and significantly upregulated in HCCs compared to their adjacent non-tumor counterpart. The analysis of differential R-values displayed three HCC subgroups, the first with LASP1 up regulation (23/55, 42%), the second with LASP-1 downregulation (19/55, 34%) and the third with similar LASP-1 expression (13/55, 24%). No clinic parameter (gender, age, tumor grading, TNM, viral hepatitis infection and background hepatic disease) correlated with the LASP-1 mRNA levels in these three subgroups.

By subdividing the HCC patients according to the gender, LASP-1 mRNA was significantly overexpressed in HCC compared to the PT tissues in female HCC patients while in males the levels were comparable. We have not yet investigated the reasons of this finding. In general, men are two to four times more often associated with HCC than women (26) and in our study the number of males and females enrolled reflected this proportion. It is known that men and women have a different risk in developing HCC since estrogens prevent while androgens promote liver cancer but the molecular mechanism of action remains unclear (27). We cannot exclude that the overexpression of LASP-1, at least at mRNA level, in the HCC tissue may be regulated by different mechanisms in males and females and that sex hormones might be involved. Note that LASP-1 is known to be upregulated in breast cancer, where it was first identified, and in ovarian cancer that are malignancies promoted by female hormonal components (9,17). Foxa1 and Foxa2 were reported as focal transcription factors for the sexual dimorphism of HCC (28). Using bioinformatics (29) we actually verified that the LASP-1 promoter could be recognized by both Foxa1 and Foxa2 in multiple sites. Whether the LASP-1 differential expression in female and male HCCs may be controlled by these two transcription factors remains a possibility and an interesting point for further investigation.

By subdividing the HCC patients in cirrhotic and non-cirrhotic HCCs, in the present work, for the first time, we found a significant LASP-1 overexpression in the HCCs developed in cirrhotic livers. Therefore, the evaluation of LASP-1 mRNA levels in cirrhotic livers (i.e. in liver biopsies) might be a useful tool to monitor the progression of the disease from hepatic cirrhosis to the HCC stage. Most of the HCCs occur in the setting of cirrhosis that is present in approximately 80–90% of HCC patients and represents the largest single risk factor and hence can be considered a premalignant condition (1). Its presence impacts survival, strongly influences treatment decisions, and clearly needs the increasingly common multidisciplinary approach to HCC management.

When we were carrying out this study, Wang *et al* provided IHC evidence for LASP-1 protein expression generally higher in HCC than in adjacent non-tumoral tissues and its expression level was associated with HBsAg and AFP levels (12). In our study, we did not find any correlation between LASP-1 mRNA expression and HBsAg and AFP, but we tested and verified in few cases that in our specimens the mRNA and protein expression of LASP-1 were comparable and generally displayed the same trend of expression. This was not obvious since it is well known that a given mRNA and the correspondent protein not necessary follow the same trend of expression. Hence we can argue that the regulatory mechanisms underlying the differential expression of LASP-1 in HCC might act at mRNA level. For IHC determinations, the protein levels were more difficult to determine and compare than mRNA levels since the localization of LASP-1 protein was nuclear, cytoplasmic or both; we believe that LASP-1 mRNA levels are better quantifiable and in this study it allowed the stratification of the cases on the basis of the gender and the presence of hepatic cirrhosis as a background liver disease. The stratification of HCC cases in more homogeneous groups defined by clinical features and molecular parameters can be of help to study and to identify targeted therapies, one of the ambitious goals of basic molecular oncology.

Concerning the molecular characterization of LASP-1 in HCC cells, with the use of MALDI-TOF mass spectrometer we found that vimentin (VIM) is a new molecular partner of LASP-1 in two undifferentiated HCC cell lines. VIM is a member of the intermediate filaments (IF) and it is important in determining the cytoskeleton structure and its changes in both physiological and pathological conditions (30). VIM also acts as signal transducer, relaying information from the extracellular matrix (ECM) to nuclei and it is an important hallmark of the EMT (epithelial-mesenchymal transition) that results in the loss of cellular adhesion and increased migratory and invasive activities of several types of tumor cells (31). It is known that the overexpression of VIM is significantly associated with HCC metastasis and it is a circulating molecular biomarker for HCC (32,33) and that LASP-1 induces TGF-β-mediated EMT transition in human colorectal cancer (34). As mentioned above, in previous studies, we demonstrated that uPA is overexpressed in HCC and that it is a responsive therapeutic target since its inhibition provoked a decrease of HCC cellular migration and invasion (25,35–37). We also demonstrated LASP-1 as a mediator of uPA in cell motility (20). With the results obtained in the present study, we can add new knowledge and hypothesize that VIM can directly interact with LASP-1 probably during the cytoskeleton dynamics necessary for the HCC cell motility. It will be of interest to assess in the future whether the mRNA expression of VIM and LASP-1 is coordinated in HCC.

For cell localization of LASP-1 protein, data in the literature point the attention to the nuclear localization of LASP-1 in some cancers (e.g. breast cancer and HCC) (17–19). Utilizing IHC and confocal immunofluorescence analysis we observed the nuclear localization of LASP-1 in HCC tissues and in human cells. Among the cases analyzed by IHC, one presented intra-hepatic recurrence (LV 144) and it showed almost exclusive nuclear localization with enhanced expression compared to the primary tumor. This could be in line with the consideration that the nuclear localization of LASP-1 may be associated with a less favorable prognosis of cancer patients (18).

In our *in vitro* studies the use of confocal immunofluorescence displayed the nuclear localization of LASP-1 both in normal human fibroblasts and in HCC derived cells, but at higher level in cancer cells as expected. To the best of our knowledge, no data on nuclear localization in the normal biological context exist as yet in the literature; therefore our observations exclude that the nuclear localization of LASP-1 may be specifically cancer-associated. LASP-1 in the nucleus might be involved in controlling gene expression possibly as a co-transcription factor and it may contribute in defining the nuclear F-actin architecture (19). Nuclear localization in human fibroblasts suggests that LASP-1 could be involved in the functions described above not only in the cancer context but also in normal conditions. Further studies are needed to better understand the functions of LASP-1 in the nucleus.

In conclusion, the present study has evidenced the upregulation of LASP-1 mRNA expression particularly in female HCCs and in cirrhotic HCCs. The identification of groups of HCC patients with shared molecular and clinical characteristics is important to set up the follow-up of the patients and to study better therapies (37). Moreover, we have identified VIM as a new molecular partner of LASP-1. Most probably, the overexpression of uPA, LASP-1 and VIM in HCC triggers the malignant ability of the HCC cells, in particular migration, because this requires the cytoskeleton remodeling. There are some anticancer drugs in current clinics that directly affect vimentin, such as silibinin and withaferin A (38,39). The finding that LASP-1 can collaborate with VIM and uPA in aggressive HCC cells may be of help in future studies of innovative therapies targeting these molecules alone or in combination or by miR-mediated negative regulation (40,41).

## Figures and Tables

**Table I:** Clinical and pathological characteristics of the studied population.

Case	Gender	Years	Grading	TNM	Background disease	HBV	HCV
LV 136	F	79	G2	NA	Cirrhosis with active chronic hepatitis	−	+
LV 185	M	66	G1	T1N0M0	Chronic hepatitis	+	−
LV 197	M	70	G2	T1N0M0	Cirrhosis with micro- and macrovescicular steatosis	−	−
LV 204	M	74	G3	NA	Active cirrhosis	−	+
LV 216	F	67	G1	NA	Cirrhosis	−	−
LV 218	M	64	G2	NA	Cirrhosis with active chronic hepatitis	NA	NA
LV 219	M	57	G1	T1N0M0	Cirrhosis with active chronic hepatitis	+	−
LV 224	M	55	G3	T3bN0M0	Cirrhosis with active chronic hepatitis	+	+
LV 225	M	49	G3	T3bN0M0	Microvescicular steatosis	−	−
LV 227	F	72	G2/G3	T1N0M0	Cirrhosis with active chronic hepatitis	−	+
LV 228	M	59	G2	T1N0M0	Active chronic hepatitis of severe level with necrosis and bridging porto-portal fibrosis (HBsAg)	+	−
LV 229	F	79	G2/G3	T3bN0M0	Cirrhosis with active chronic hepatitis	−	−
LV 232	M	76	NA	NA	Cirrhosis with chronic hepatitis	−	+
LV 235	F	82	G3	T2N0M0	Cirrhosis with active chronic hepatitis	−	+
LV 236	F	76	G1	T1N0M0	Cirrhosis with active chronic hepatitis	−	+
LV 237	M	68	G2/G3	T1N0M0	Mildly active chronic hepatitis	−	+
LV 240	M	71	G3	T3bN0M0	Active chronic hepatitis with necrosis and bridging and portal fibrosis	+	−
LV 241	F	38	G2	T3N0M0	Reactive hepatitis	+	−
LV 242	F	63	G2	T2N0M0	Active chronic hepatitis with focal fibrosis and with bridging porto-portal fibrosis	−	+
LV 262	M	73	G2	NA	Mild hepatitis	−	−
LV 268	F	68	G1	T1N0M0	Cirrhosis with active chronic hepatitis	+	−
LV 273	M	73	G2	T1N0M0	Cirrhosis with active chronic hepatitis and mild macro- and microvescicular steatosis	−	+
LV 274	F	81	G2	T1N0M0	Mildly active chronic hepatitis with micro- and macrovescicular steatosis (30% of parenchyma)	−	−
LV 276	M	72	G2	T1N0M0	Cirrhosis with active chronic hepatitis	−	+
LV 277	F	75	G2	NA	Chronic hepatitis	−	−
LV 278	M	72	G2	NA	Cirrhosis	−	+
LV 279	M	70	NA	NA	Chronic hepatitis	−	+
LV 280	F	74	G2/G3	T1N0M0	Cirrhosis with active chronic hepatitis	−	+
LV 281	M	74	G1	NA	Normal parenchyma	−	−
LV 283	M	78	G2	T1N0M0	Mildly active chronic hepatitis	+	−
LV 284	M	76	G2	T1N0M0	Active chronic hepatitis	+	−
LV 285	M	77	G2	T2N0M0	Active chronic hepatitis with moderate/severe necrosis	−	+
LV 286	M	69	G3	T4N0M0	Active cirrhosis	−	+
LV 287	M	63	G2	T2N0M0	Active cirrhosis	−	−
LV 288	F	64	G2	T1N0M0	Active cirrhosis	−	−
LV 290	M	65	G2/G3	T1N0M0	Active cirrhosis	+	−
LV 291	M	69	G1	NA	Hepatitis	+	+
LV 292	M	66	G3	NA	Active cirrhosis	−	+
LV 293	M	61	G3	NA	Active cirrhosis	+	+
LV 294	M	61	G2	NA	Hepatitis	−	−
LV 295	M	72	G3	NA	Active cirrhosis	−	−
LV 296	M	67	G3	NA	Active cirrhosis	+	−
LV 297	M	73	G3/G4	NA	Hepatitis	+	−
LV 302	F	61	G2	NA	Cirrhosis	−	−
LV 303	M	76	G3/G4	NA	Cirrhosis	−	−
LV 304	M	70	G2	NA	Cirrhosis	−	+
LV 305	F	76	G3	NA	Hepatitis	−	+
LV 306	M	48	G3	NA	Hepatitis	−	+
LV 307	M	64	G2	NA	Steatosis	−	−
LV 308	M	73	G2	NA	Active cirrhosis	−	−
LV 310	F	71	G1	NA	Active cirrhosis	−	+
LV 317	F	69	G1	NA	Cirrhosis	+	−
LV 318	F	71	G2	NA	Cirrhosis with ECA	−	+
LV 323	F	78	G3	NA	Hepatitis	−	+
LV 325	F	65	G3	NA	Chronic hepatitis with ECA	−	+

NA, not applicable.
